# Dietary Docosahexaenoic Acid (22:6) Incorporates into Cardiolipin at the Expense of Linoleic Acid (18:2): Analysis and Potential Implications

**DOI:** 10.3390/ijms131115447

**Published:** 2012-11-21

**Authors:** Colin H. Cortie, Paul L. Else

**Affiliations:** Metabolic Research Centre (in IHMRI), School of Health Sciences, University of Wollongong, Wollongong, NSW 2522, Australia; E-Mail: pelse@uow.edu.au

**Keywords:** phospholipids, fatty acids, mitochondria, peroxidation, apoptosis, cancer, tumor

## Abstract

Cardiolipin is a signature phospholipid of major functional significance in mitochondria. In heart mitochondria the fatty acid composition of cardiolipin is commonly viewed as highly regulated due to its high levels of linoleic acid (18:2*n* − 6) and the dominant presence of a 4×18:2 molecular species. However, analysis of data from a comprehensive compilation of studies reporting changes in fatty acid composition of cardiolipin in heart and liver mitochondria in response to dietary fat shows that, in heart the accrual of 18:2 into cardiolipin conforms strongly to its dietary availability at up to 20% of total dietary fatty acid and thereafter is regulated. In liver, no dietary conformer trend is apparent for 18:2 with regulated lower levels across the dietary range for 18:2. When 18:2 and docosahexaenoic acid (22:6*n* − 3) are present in the same diet, 22:6 is incorporated into cardiolipin of heart and liver at the expense of 18:2 when 22:6 is up to ~20% and 10% of total dietary fatty acid respectively. Changes in fatty acid composition in response to dietary fat are also compared for the two other main mitochondrial phospholipids, phosphatidylcholine and phosphatidylethanolamine, and the potential consequences of replacement of 18:2 with 22:6 in cardiolipin are discussed.

## 1. Introduction

Cardiolipin (CL) is the only prevalent di-phospholipid present in mammalian membranes [1–4] and is primarily found in the inner leaflet of the inner mitochondrial membrane where it is synthesized [1]. As a consequence of its structure CL possesses four rather than the usual two fatty acids and may be viewed either as two phosphatidic acid molecules joined together by glycerol, or as a phosphatidic acid joined to a phosphatidylglycerol molecule, or even as two phosphatidylglycerol molecules sharing the same glycerol molecule. As CL is found almost exclusively in mitochondria it has been called the signature phospholipid of mitochondria [2]. CL has important roles in oxidative phosphorylation and in triggering mitochondrial-induced apoptosis [2,3,5]. These roles in turn are linked to CLs ability to bind mitochondrial proteins required for ATP production [4,6] and to peroxidise in interactions with cytochrome c [7].

Phospholipids vary extensively in their acyl composition containing a mixture of saturated (SFA), monounsaturated (MUFA) and polyunsaturated (PUFA) fatty acids [8], yet most phospholipids (>99%) in mammalian membranes avoid multiple PUFAs on the same phospholipid molecule [8,9]. This seems not to apply to CL that in cardiac mitochondria has a high PUFA content with a tetra-linoleic acid (4×18:2*n* − 6) molecular species being its most prevalent molecule. This tetra linoleic acid species of CL has been found to associate with the mitochondrial ADP/ATP carrier more close than a tetra-palmitoyl (4×16:0) species [10] and to have a higher affinity for cytochrome *c* in comparison to CL molecules with high levels of oleic (18:1), stearic (18:0) and palmitic acid (16:0) respectively [11]. High levels of the 4×18:2 species of CL in cardiac mitochondria and high levels of 18:2 generally are described in mitochondria isolated from other mammalian tissues. For example, Schlame *et al*. [12] reported that 4×18:2 CL comprises 80% of all CL in human heart, 70%–77% in dog heart, 41%–77% in rat heart and 50% in cow heart, while Han *et al*. [13] reported that this same species of CL comprises 42% of CL in rat heart and 52% in rat liver. Other groups have reported the 18:2 content of CL in liver and brain as lower than 60%, meaning that the 4×18:2 molecular form of CL may not be as common in some of these tissues [14–16].

Although high 18:2 content is seemingly preferred in CLs interactions with proteins [10], this high PUFA content coupled with CLs potential exposure to free radical oxygen species (ROS) produced in the mitochondria put this phospholipid at high risk of oxidative damage [2]. Peroxidation at bis-allylic carbons (*i.e.*, –CH=CH-CH_2_-CH=CH–) present in PUFAs is the primary form of oxidative damage that occurs in phospholipids. The reason for this is that hydrogen atoms bound to bis-allylic carbons have weaker bond energies due to the presence of the surrounding double bonds, making them more prone to abstraction by free radicals [17]. Therefore, the more bis-allylic carbons, the higher the number of weaker bound hydrogen atoms the more peroxidisable the fatty acid [18]. Although 18:2 is the least unsaturated of the PUFAs, the fact that there are up to four of these fatty acids packed together on the same molecule makes CL highly likely to peroxidise in comparison to other mitochondrial phospholipid classes. Peroxidation of CLs PUFAs is likely to decrease its affinity for protein binding, disrupt the structure of the inner mitochondrial membrane and produce a number of cytotoxic products, with CL peroxidation identified as an early event in the apoptotic cascade [11,19–22]. Therefore, changes in CLs PUFA content could be expected to affect both its interaction with proteins and susceptibility to peroxidation, modifying its function in mitochondria. Such changes are thought to be unusual as one of the widely held beliefs about mammalian CL fatty acid composition is that it is highly regulated, primarily due to its high levels of 18:2 and the prevalence of a 4×18:2 molecular species in cardiac mitochondria and an apparent resistant to dietary manipulation [12]. Here we review the evidence that, contrary to popular belief, CLs PUFA composition is amenable to change dependent upon the fat composition of the diet, particularly the presence of docosahexaenoic acid (22:6*n* − 3) that will readily replace 18:2 when present in the diet at up to 20% of total fatty acid.

## 2. Cardiolipin Response to Dietary Fat Composition

### 2.1. Dietary Trials Investigating Cardiolipin in Animal Models

It has been known for some time that dietary fats affect phospholipid composition [23] and CL seems to be no exception. The fatty acid composition of CL has been reported in numerous studies examining the effects of various dietary fatty acids on the phospholipid composition of heart and liver mitochondria. Most (~75%) of these studies have used rats [15,16,23–38] and most others, mice [39–43]. These studies supply the raw data used in the present study for an analysis of the 18:1, 18:2 and 22:6 content of CL in heart and liver against changes in dietary fat.

The primary focus of many of these studies has been PUFA, that cannot be produced in mammals unlike SFA and MUFA that can be formed *de novo* from basic carbon sources [44]. The sources of dietary SFA, MUFA and PUFA used in these studies have been in the form of oils commonly eaten by humans including fish, linseed, corn, olive, rapeseed and soybean, although one study used crocodile oil [41] (see Table 1). The majority of the studies predate the use of mass spectrometry, instead using combinations of thin layer chromatography or high-pressure liquid chromatography together with gas chromatography. One limitation of these techniques (*versus* a lipidomics approach) is that they cannot readily determine the abundance of specific phospholipid molecular species within each phospholipid class. However, these studies do provide the total fatty acid composition of the various CL molecular species.

### 2.2. Regulator-Conformer Paradigm

In order to better understand the relationship between CL composition and the fatty acid composition of the diet we analysed all available data in accordance with the conformer-regulator paradigm (often used in comparative physiology), plotting the level of a fatty acid in the diet (*i.e.*, 18:1, 18:2, 22:6) against its level or that of another fatty acid (e.g., 22:6) in CL. If the fatty acid composition of CL conformed perfectly to its dietary composition (*i.e.*, is a conformer) a slope of 1 is produced (when the proportion of a fatty acid in the diet is plotted against its level in CL) but if fatty acid composition is perfectly regulated (*i.e.*, not influenced by diet) the slope is zero [46]. In this model, slopes not significantly different from zero would indicate regulation whereas those significantly greater than zero but below 1 would indicate weak to strong dietary conformity, and slopes of 1 or more would indicate very strong, active dietary conformity. Previous work using this model has shown that total SFA, MUFA and PUFA content are likely to show a regulator pattern, but within PUFAs, the omega-6 (n-6) and omega-3 (n-3) fats such as 18:2 and 22:6 respectively, tend to show varying levels of dietary conformity, particularly at low dietary levels where presumably their essential dietary nature is displayed [47,48].

As the primary aim of this study was to examine CL remodeling in relationship to dietary fat consumption in healthy animals, a small number of exclusion criteria needed to be applied: 18:2 is the most readily consumed PUFA in the human diet (~42% of fatty acids consumed [49]) and an essential fatty acid in mammals. Therefore our analysis excluded any diets containing less than 2% of 18:2 as a proportion of total dietary fatty acid. Based on the work of Owen *et al.*[50], in order to allow adequate time for phospholipid remodeling to occur, any dietary interventions of two weeks or less were excluded. Where dietary levels of eicosapentaenoic acid (20:5*n* − 3 or EPA) were greater than 10%, or in the case comparing 18:2 versus 22:6 in the same diet (Figure 2) where 20:5 levels exceeded those of 22:6, these diets were excluded due to the confounding effect of 20:5 conversion through to 22:6. In the entire analysis the only study excluded as a gross outlier was that of Berger *et al.*, 1992 [42] whereas all other studies, including another Berger [40] study were included. Although many studies reported SFA in low levels in CL, presumably due to the presence of immature CL (see discussion in section 3), SFA content was not examined, as not all studies reported SFA’s shorter than 18:0. In the case of the omega-3 fatty acid linolenic acid (18:3), although it is consumed at moderate levels in the diet [49], little if any appears in most phospholipids [8] including CL [12], presumably due to its conversion through to longer chained omega-3 fats. The omega-6 fatty acid arachidonic acid (20:4) was not included in the present analysis but has been reported in CL at levels ranging from 0–12% [23,26,40]. Where different feeding periods were used in the same study the period closest to four months was chosen since it represented an average common feeding period across the studies. Diets with unspecified 22:6 from maternal sources or high proportions of trans-fats where excluded from the analysis.

Line fit and slopes in all figures were determined using segmental linear regression with an unconstrained line intercept available in GraphPad Prism® Version 6 (Graphpad Software: La Jolla, CA, USA, 2012). All statistical analyses were performed using the same software package and slope values are reported as slope ±95% confidence interval.

### 2.3. Unsaturated Fatty Acid Composition of Cardiac Cardiolipin Against Dietary Levels

**Figure 1:**
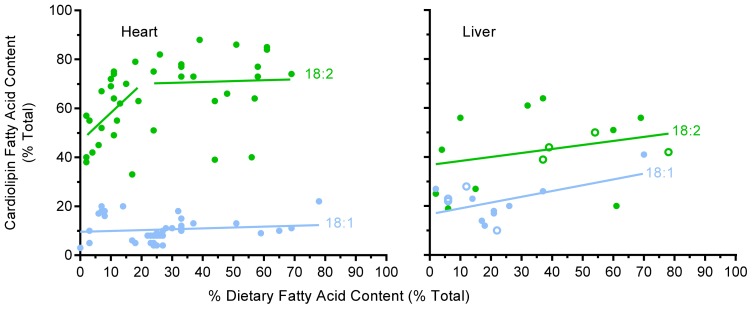
The relative content of 18:1 and 18:2 of cardiolipin in heart and liver against their dietary levels. Values for heart are for rat (●) and those for liver are for rat and mice (○). For heart, a biphasic pattern of incorporation of 18:2 into CL was apparent (also present with mice values added) with a rapid initial conformer uptake between 2%–20% of dietary 18:2 and thereafter a relatively constant, regulated level. No biphasic trend was apparent for 18:1 in heart or 18:1 and 18:2 in liver. Data for heart came from the following studies [15,16,23,24,26–29,33,35–38,45,51] and that for liver from [15,24,33,35,40,41,52]. Diets with greater than 3% 22:6 or greater than 10% EPA were excluded from the analysis due to effects on 18:2 (see Figure 2) and conversion of 20:5 through to 22:6.

Oleic (18:1), linoleic (18:2), and docosahexaenoic (22:6) acids are the three most commonly reported unsaturated fatty acids present in CL [15,16,24,26–30,32,33,39–41,43,45]. Figure 1 shows the percentage of 18:1 and 18:2 in CL against their percentage in the diet in heart and liver. The incorporation of 18:2 into cardiac CL shows a distinctive biphasic pattern with a rapid rise in CL 18:2 levels from 2%–20% of dietary fat indicating a conformer pattern as indicated by a slope of 1.17 ± 1.10 (*p* = 0.038). At these lower levels of dietary 18:2 intake, CL seems to be actively accruing 18:2 into its structure, as would be predicted from the high 18:2 content generally reported for CL. At dietary levels where 18:2 is above 20% (range 21%–68%) of dietary fat, the incorporation of this fatty acid reduces to a regulated pattern with a slope of 0.036 ± 0.524, leveling off with ~70% of CL fatty acid being 18:2 (or ~65% when rat and mouse values are combined), supporting the common belief that 18:2 levels in CL is largely a regulated phenomenon. In the case of 18:1, no significant trend was apparent with a slope of 0.036 ± 0.094 (*p* = 0.886) across a wide dietary range (0–78%), indicating a regulated pattern with 18:1 comprising around ~10% of cardiac CL fatty acid content.

**Figure 2:**
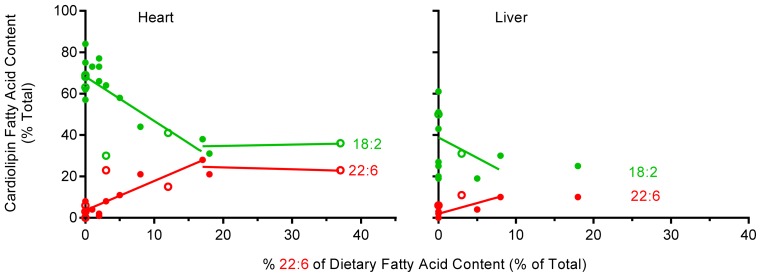
The relationship between the dietary availability of 22:6 and 18:2 and cardiolipin content of the same fatty acids in heart and liver of rat (●) and mice (○). Data for both organs indicate a biphasic relationship with CL 22:6 content increasing, and 18:2 content decreasing at dietary 22:6 levels between 0–20% and thereafter stabilizing. Data used included: control diet [36], sunflower oil and tuna oil [23], diet 1 and 4 [39], 4 month time point [45], high 18-3 diet, long chain n-3 diet [43], control, high 22:6 [33], soy oil and crocodile oil [41], sardine oil, corn oil [15], 18:2 and long chain n-3 [28].

Analysis of 22:6 levels in heart CL (Figure 2) shows that when 22:6 is absent in the diet a small amount of 22:6 (2%–8%) is still present in CL, demonstrating conversion such as that of 18:3 by the liver (the primary 22:6 synthesizing organ in mammals [53]) and its incorporation into heart CL. When 22:6 is present in the diet at levels of up to 20% of total fatty acid it is readily incorporated into CL, indicating a strong active conformer pattern (as found for 18:2) with a slope of 1.43 ± 0.58 (*p* < 0.0001). Although 22:6 was examined over a more attenuated range (0–37%) than 18:2, once the dietary levels of 22:6 were >20% of total dietary fatty acid, the pattern is suggestive of a regulated level as indicated by the plateauing of 22:6 levels at ~20% of fatty acid CL content. Therefore 18:2 and 22:6 seem to be readily incorporated into heart CL when their levels in the diet are up to 20% of total dietary fatty acid, indicating a strong active conformer pattern, but at higher dietary levels a regulated pattern is suggested, whereas 18:1 shows a regulated pattern throughout a wide range of dietary availability.

### 2.4. Unsaturated Fatty Acid Composition of Hepatic Cardiolipin against Dietary Levels

Fewer studies have examined CL acyl remodeling in liver compared to heart, but what is available [24,31,33,40,41] is shown in Figure 1 for 18:1 and 18:2 and in Figure 2 for 22:6 (both figures use combined species data). In general, the level of 18:1 in liver is higher (at ~20%), and 18:2 and 22:6 levels are lower than those in heart. Presumably 18:1 is acting as an alternative for some of the 18:2 and 22:6 fatty acids in liver, with some studies reporting 18:1 levels as high as 55% in liver CL [24,31,35]. There was no clear trend for 18:1 incorporation into liver CL other than towards a very mild, but not significant accrual (with a slope of 0.237 ± 0.266; *p* = 0.076) throughout the dietary range examined (2%–70%) with overall 18:1 levels seemingly regulated. Similarly, 18:2 showed little indication of any strong trend to incorporate into liver CL at either low or high dietary 18:2 intakes (Figure 1) with a slope of 0.164 ± 0.352; (*p* = 0.33) across a broad dietary range, again indicating an essentially regulated phenomenon. Although there is little data for 22:6 in liver CL (Figure 2), what is available suggests a conformer pattern when 22:6 levels are below 10% of total dietary fatty acid with a slope of 1.04 ± 0.85 (*p* = 0.02).

### 2.5. The Effect of Dietary 22:6 on the Incorporation of 18:2 into Heart and Liver CL

Heart CL clearly accrues both 18:2 and 22:6, and liver CL accrues 22:6 at low dietary levels, but how do dietary levels of 22:6 influence levels of 18:2 in CL? Here we examine all studies that incorporated both 22:6 and 18:2 into the same diet and measured heart [15,23,28,33,37,39,41,43,45] and liver [15,31,33,41,42] CL fatty acid composition. The results of this analysis are shown in Figure 2. When 22:6 was between 0–20% of dietary fat, the 18:2 content of CL ranged from 1%–58% with an average of 21% and no statistical relationship found between the dietary levels of these two fatty acids. For example, in heart when 22:6 was at its highest level in the diet (37%) the level of 18:2 was also one of the highest of any of the diets (56%) indicating that changes in 18:2 levels in CL are not simply due to 22:6 displacing 18:2 in the diet.

Owing to the more limited number of studies available that included both 22:6 and 18:2 in the same diet, the analysis includes data for both rat and mouse tissues. Figure 2 clearly shows the deposing influence of 22:6 on 18:2 levels in cardiac CL at 22:6 levels of up to 20% of total dietary fatty acid. At dietary levels where 22:6 is less than 20% of total fatty acids, 22:6 incorporates into cardiac CL (slope 1.43 ± 0.58; *p* = 0.0001) whilst at the same time in the same cardiac tissue the levels of 18:2 in CL decreases (slope −2.14 ± 1.24; *p* = 0.002). At higher 22:6 levels (above 20% of fatty acids), 18:2 appears to plateau at just under 40% of total fatty acids in heart CL which is below its “normal level” of ~70% (as shown in Figure 1), whereas 22:6 plateaus at just above ~20% of total fatty acid making up most of the difference (Figure 2). Although somewhat less convincing, due to the smaller amount of data available, liver CL also appears to accrue 22:6 at the expense of 18:2 (slope 1.041 ± 0.846; *p* = 0.022), when it is available at low levels in the diet (Figure 2). Furthermore, although based on a single study [33], the current evidence suggests that liver CL does not accrue more 22:6 once its levels are above 10% of total fatty acid in the diet. At this point 18:2 plateaus at slightly over ~20% of CL fatty acid content, down on the normal ~30%–60% (see Figure 1), with 22:6 at ~13% of CL fatty acid making up a large proportion of the difference. Therefore CL in heart and liver maintain 18:2 levels at ~70% and 50% respectively, and in the case of heart will readily accrue 18:2 from the diet when this fatty acid is at lower levels (<20%). However, when 22:6 is available in the diet it is incorporated at the expense of 18:2 until 22:6 makes up ~20% of CL fatty acid content in heart and ~13% in liver, after which both 18:2 and 22:6 levels seem to remain relatively constant *i.e.*, regulated.

### 2.6. A Comparison of 22:6 Incorporation into Mitochondrial Phospholipids

**Figure 3:**
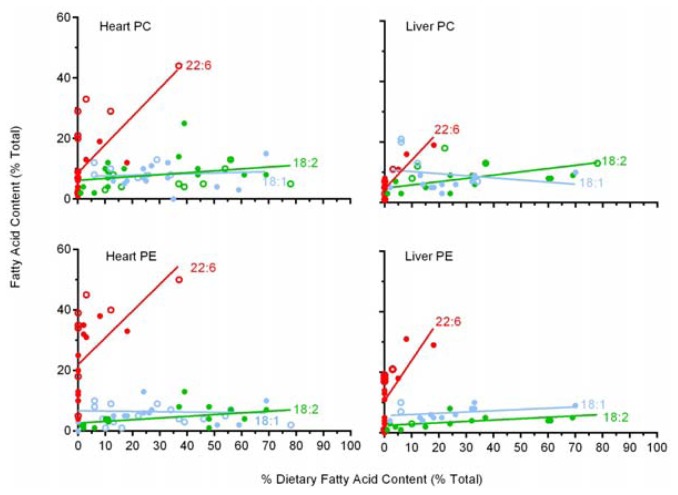
The relative content of 18:1, 18:2 and 22:6 in phosphatidylcholine (PC) and phosphatidylethanolamine (PE) of heart and liver mitochondria against their respective level in the diet. Each figure includes both rat (●) and mouse (○) data. The incorporation of 18:2 into PE of both heart and liver show slight dietary conformity whereas 22:6 in both PC and PE of both heart and liver demonstrate strong dietary conformity throughout the dietary range examined. All other fatty acids in PC and PE of liver and heart demonstrated a relatively regulated content across the broad dietary range of fatty acids examined. Data used for heart PC was from [15,23,24,27,28,30,33,34,36–41,43], for heart PE from [24,25,39,40], for liver PC from [15,24,26,31,33,40,41] and for liver PE from [15,24,26,31,33,40,41]. Data excluded from the analysis included diets that contained higher 20:5 than 22:6.

Although functionally important, CL is not the major phospholipid class in mitochondria, comprising ~15% of mitochondrial phospholipids. The two major mammalian mitochondrial (and cell) phospholipids are phosphatidylcholine (PC) and phosphatidylethanolamine (PE), which makeup ~45% and ~35% of total mitochondrial phospholipid respectively [54]. Since some of the studies [11,21,22,25,26,28,29,31,32,34–41,48] examined here included data for these two major phospholipids we have included a brief analysis of 18:1, 18:2 and 22:6 incorporation into these two major phospholipids in heart and liver mitochondria. This analysis is also useful as most studies report tissue responses rather than mitochondrial responses to variations in dietary fat. The results of this analysis are shown in Figures 3.

The incorporation of 18:1 and 18:2 into PC and 18:1 into PE of cardiac mitochondria, as shown in Figure 3, indicates that there are no significant trends in the incorporation of these fatty acids into these two phospholipids across a broad range of dietary supplementation (2%–78%), indicating a regulated pattern in heart. The incorporation of 18:2 into cardiac PE shows a weak but significant conformer trend with increasing levels across the same dietary range with a slope of 0.056 ± 0.051 (*p* = 0.034). In contrast, 22:6 in both PC and PE in heart mitochondria display a strong conformer trend with slopes of 0.93 ± 0.45 (*p* = 0.0003) for PC and 0.89 ± 0.58 (*p* = 0.005) for PE when 22:6 levels were up to 38% of dietary fatty acid.

In liver mitochondria the same pattern is present, namely the incorporation of 18:1, 18:2 into PC and 18:1 into PE showing no significant trends in their incorporation into these two phospholipids across a broad dietary range. Liver mitochondrial PE shows a weak conformer uptake for 18:2 (slope 0.045 ± 0.038; *p* = 0.024) but both liver PC and PE both show a strong dietary conformer pattern for 22:6 uptake with slopes of 0.95 ± 0.32 (*p* < 0.0001) for PC and 1.33 ± 0.79 (*p* = 0.0025) for PE across a dietary range of 22:6 of up to 20%.

Therefore the general pattern in liver and heart PC and PE was for 18:1 to be regulated in both classes of both tissues, for 18:2 to be regulated in PC heart and liver but a mild conformer effect in PE, and for 22:6 to be a strong conformer to dietary levels in both classes and tissues. As mitochondria membranes represent a significant portion of cell phospholipids and CL, PC and PE are the most common mitochondrial phospholipids, increases in their 22:6 content presumably accounts for a portion of the well-known [50] dietary induced increase in 22:6 levels in total membrane phospholipids.

## 3. Consequences of Cardiolipin Composition

The present analysis of a comprehensive compilation of studies reporting changes in CL fatty acid composition in response to dietary fats shows that the accrual of 18:2 into cardiac CL occurs readily. The analysis indicates a strong conformer pattern when dietary levels of 18:2 are between 2%–20% of total dietary fatty acid, with higher dietary availability of 18:2 showing regulated levels. In liver, no trend was evident for 18:2 incorporation into CL across a broad range of dietary 18:2 intakes, indicating a regulated level of 18:2 into CL of liver mitochondria. When 22:6 and 18:2 are present in the same diet, and 22:6 is between 0–20% of total dietary fatty acids, the data indicates that CL preferentially incorporates 22:6 into its structure at the expense of 18:2. This preferred incorporation of 22:6 over 18:2 seems to be present in both heart and liver. This ability of 22:6 to displace 18:2 has previously been noted in phospholipids of heart. For example, in a dose response study of dietary 22:6 (0–32%) examining myocardial phospholipids, Owen *et al.*[50] found that 22:6 replaced 18:2, and to a lesser extent 18:1. Other studies comparing diets low and high in 22:6 have noted similar trends in cardiac phospholipids [39,41] and CL specifically [15,24,34,41].

The present analysis found that not only will 22:6 replace 18:2 in CL, but also that this replacement will occur only at dietray 22:6 levels of up to ~10% and 20% in liver and heart respectively and against high dietary background levels of 18:2 (of up to 56%). The strength and veracity of these relationships is highlighted when considered against the highly varied dietary backgrounds used in the studies that produced the results. Exclusions were limited to only diets deficient in 18:2, took place for no longer than two weeks or had high 20:5 backgrounds when examining 22:6. Based on our analysis, we have shown that CL is a molecule with a preference for 18:2 but an even greater fondness for 22:6 (when 22:6 is available in the diet at up to 10% or 20% of total fatty acid for liver and heart respectively). Dependent upon its availability in the diet, cardiac CL will accrue 22:6 up to a maximum of approximately 20% and liver CL up to 13% of its total fatty acid content.

The selectivity of 22:6 over 18:2 in CL and the preferential incorporation of these two fatty acids over others is likely due to the complex nature of CL synthesis and remodeling. CL is synthesized from the condensation of a PG molecule with cytidine diphosphate diacylglycerol resulting in what is commonly referred to as immature CL that has a mixed acyl content and is then remodeled to produce mature CL [2]. The selected incorporation of fatty acids during remodeling was recently investigated by Kiebish *et al.*[55], who reported that only 1-5% of the fatty acids incorporated into CL during the remodeling process come from PC and PE, with this process having low acyl selectivity [55]. By far the majority of fatty acids are drawn from the pool of acyl CoA fatty acids and this incorporation is highly selective [55]. Interestingly, heart acyltransferases were reported to have 1.3 times the specificity for 22:6 over 18:2, which in turn had specificity many times higher than that of any other fatty acid. In contrast, liver acyltransferases had a far higher specificity for 18:2 over 22:6 or any other fatty acid [55]. The specificities of these acyltransferases, and tissue differences are likely to explain the underlying mechanism by which 22:6 replaces 18:2 in heart CL but does not explain the response in liver. This remodeling of CL is similar to that of PC and PE in that all three classes are dependent upon the activity and specificities of various acyltransferases [56].

Based on the roles CL performs in the mitochondria, the functional consequences of accruing 22:6 into the structure of CL are likely to be significant. Three studies have examined the effect of 22:6 incorporation into CL on mitochondrial oxidative phosphorylation. These studies reported either no change [39] or a decrease in activity [15,38]. More recent work in this area has shown a similar trend of dietary 22:6 either decreasing or not affecting mitochondrial respiration [57,58]. It is possible that 22:6 in CL might increase inner mitochondrial membrane fluidity and permeability to protons, decreasing the efficiency of oxidative phosphorylation suggesting that 18:2 is a preferred fatty acid for CL-protein interactions [10]. The incorporation of 22:6 over 18:2 into CL will increase its susceptibility to oxidative damage (peroxidation), which is an important mechanism in both cell health and diseases [59–61]. The peroxidisability of 22:6 is about 5 times that of 18:2 [62]. So the incorporation of 22:6 at the expense of 18:2 at up to ~20% of CLs acyl content will more than double CLs likelihood of undergoing oxidative damage. CL has been shown to be more susceptible to peroxidation than other phospholipid classes both *in vitro*[63] and *in vivo*[20] due to its high level of 18:2 and proximity to ROS production and the presence of 22:6 will accentuate this susceptibility. The remodeling of CL with increased 22:6 via the presence of a lyso CL acyltransferease (ALCAT1) has also been implicated in pathological remodeling reminiscent of type-2-diabetes, although numerous other changes were also associated due to the presence of this particular acyltransferase [64].

The natural peroxidation of CL is likely coupled to its binding to the haem-containing cytochrome *c*[7,65,66] and may be related to the transfer of CL from the inner to the outer mitochondrial membrane during apoptosis [67]. Cardiolipin peroxidation is thought to be an early event in mitochondrial-induced apoptosis related to cytochrome c release, production of cytotoxic products and disruption of the inner and outer mitochondrial membranes [11,19,20]. Therefore, the replacement of 18:2 by 22:6 would be expected to increase mitochondrial susceptibility to oxidative damage and apoptosis [68]. In addition, the products formed during peroxidation of omega-3 PUFA such as 22:6 are proposed to be more reactive, and potentially disruptive, than those produced from omega-6 PUFA such as 18:2 [69]. However, the fact that CL accrues 22:6 in place of 18:2 increasing its peroxidative potential clearly indicates that under normal dietary circumstances this is not a problem as peroxidation of mitochondrial phospholipids would seem to offer a selective property if it was disadvantageous. The increased presence of 22:6 in the cardiac and skeletal muscle membrane has been linked with improved oxygen efficiency of muscular contractions and fatigue resistance [70]. Yet these changes are likely underpinned by interactions occurring between phospholipids and proteins at the molecular level within the membrane. This is where the acyl composition of phospholipids can influence membrane proteins by changing factors such as; membrane fluidity, intramembrane pressure gradients and dipole potentials [71]. The accrual of 22:6 into phospholipids in a conformer fashion probably reflects the essential dietary nature of omega-3 generally and the readiness of tissues to use the preformed fatty acid if available. Yet the precise benefits are again likely to be found in fundamental properties that we do not fully understand and are particular to different membranes operating in a variety of different tissues.

One highly speculative area where changes in CL acyl composition might be of some potential therapeutic benefit is in the case of breast cancer treatment. The presence of 22:6 in the diet appears to sensitize this tissue to apoptosis when given alone or alongside conventional cancer treatments [72–75]. The incorporation of 22:6 into the phospholipids of mammary tumors in rats seems to occur readily and shows a conformer pattern (comparing plasma to tumor phospholipid 22:6 levels [76]) similar to cardiac CL at low 22:6 levels. This ready incorporation of 22:6 into tumor phospholipids is likely due to tumor growth, and where examined, CL seems as effective at incorporating 22:6 compared to other phospholipids [68]. The composition of CL in normal tumor tissue compared to parent tissue often shows a profile that is abnormally elevated in SFA consistent with immature CL before 18:2 remodeling [77,78]. Interestingly, lymphocytes with CL containing more saturated fatty acids (16:0) have also been found to proliferate more rapidly [79]. The high SFA in CL is likely related to common disturbances in cancer mitochondria that see’s these cells utilize aerobic glycolysis in a phenomenon commonly referred to as the Warburg effect [80]. Cancer cells also show a decreased sensitivity to apoptosis [81] and based on the potential importance of CL peroxidation in apoptosis, it has been suggested that the low PUFA content of cancer cells inhibits the peroxidation-related mechanism of apoptosis [22]. Tentatively, it might suggested that diets that facilitate 22:6 incorporation into CL (and other phospholipids) of cancer cell mitochondria might make these tumors more susceptible to apoptosis and radiation targeted tissue destruction.

## 4. Conclusions

The best-known CL molecular species is the tetra-linoleic (4×18:2) species that dominates in mammalian heart mitochondria. Familiarity with this molecule, the high level of 18:2 associated with CL and the results of a few early dietary studies produced a view that the acyl composition of CL is largely regulated and less amenable to change based on variation in dietary fat composition compared to other phospholipids [12]. However, our analysis of data from many different studies indicates that CL is amenable to change in dietary fat composition and that 18:2 accrual into cardiac CL occurs primarily at up to 20% of dietary fatty acid and thereafter levels are regulated at ~70% of CL fatty acid content. Whereas in liver there is no indication of accrual at lower 18:2 dietary levels and overall levels of 18:2 in liver CL are lower. The analysis clearly indicates that when 18:2 and 22:6 are present in the diet together, that the biochemistry of the cell favors the incorporation of 22:6 over 18:2 when 22:6 is present in the diet at levels of up to 20% in heart and also possibly in liver mitochondria (at up to 10%). The incorporation of 22:6 into mitochondrial CL, PC and PE most likely accounts for a large portion of the well-known capacity of the diet to increase the 22:6 content in total cellular membrane phospholipids. Finally, based on the importance of CL in cellular respiration and in mitochondrial-mediated apoptosis, the replacement of 18:2 with the more peroxidisable 22:6 may result in changes in the properties of CL and therefore mitochondria.

## Figures and Tables

**Table 1:** Details of species, sources of fat, measurement method and feeding period of studies examined.

Reference	Source of fat	Measurement method	Dietary fat (*w*/*w*)	Feeding period (weeks)
Rats				
Yamaoka *et al*., 1988 [15]	Corn oil, sardine oil	TLC, GC	20%	4
McGee *et al*., 1996 [16]	Not stated	TLC, GC	20%	4
Charnock *et al*., 1986 [23]	Sunflower oil, tuna, vegetable oil	TLC, GC	4% or 16%	60
Astorg *et al*., 1991 [24]	Sunflower seed oil, linseed oil	HPLC/GC 10%	20	
Ikeda *et al*., 1996 [26]	Safflower oil, perilla oil, palm oil, ethyl 20:4	TLC/[15]GC	10%	3
Innis and Clandinin, 1981 [27]	Soya-bean, rapeseed	TLC, GC	20%	5
Jahouvey *et al.*, 1990 [28]	Palm oil, olive oil, sunflower oil, linseed oil, menhaden oil	HPLC, GC	15%	4
Kramer, 1980 [29]	Corn oil, zephyr oil	TLC, GC	20%	16
Novak *et al*., 2006 [30]	Standard chow diet	TLC, GC	3.5%	9
Power *et al*., 1994 [31]	Coconut oil, olive oil, safflower oil, menhaden oil	TLC, GC	2% or 20%	10
Taniguchi *et al*., 1993 [33]	20:5 and 22:6 methylesters	TLC, GC	15%	2
Yamaoka *et al*., 1990 [34]	Corn oil, sardine oil	TLC, HPLC, GC	20%	2
Hoy and Holmer, 1990 [35]	Marine oil, olive oil, sunflower seed oil	TLC, GC	20%	10
Charnock *et al*., 1984 [36]	Sunflower seed oil, sheep kidney fat	TLC, GC	4%, 12%	20
Charnock *et al*., 1991 [37]	Sunflower, fish oil	TLC, GC	16%	44
Robblee and Clandinin, 1984 [38]	Beef tallow, soybean oil	TLC, GC	7%, 7.5%, 21% or 23%	2
Lee *et al.*, 2006 [45]	Soybean oil, fish meal, soybean meal, alfalfa meal, corn meal	TLC, GC	4%	16
Mice				
Croset and Kinsella, 1989 [39]	Ethyl esters of 18:2, 22:6	HPTLC, GC	10%	2
Berger and German, 1990 [40]	Safflower oil, free fatty acids of 18:2, 20:5	HPTLC, GC	2%	2
Watkins *et al*., 2001 [41]	Crocodile oil, soybean oil	TLC, GC	7%	13
Hussein *et al*., 2009 [43]	Coconut oil, safflower oil, flaxseed oil	HPLC, MS	10%	17

TLC: thin layer chromatography; GC: gas chromatography; HPLC: high pressure liquid chromatography; MS: mass spectrometry.
